# A molecular survey based on eDNA to assess the presence of a clown featherback (*Chitala ornata*) in a confined environment

**DOI:** 10.7717/peerj.10338

**Published:** 2020-12-17

**Authors:** Maslin Osathanunkul, Toshifumi Minamoto

**Affiliations:** 1 Research Center in Bioresources for Agriculture, Industry and Medicine, Department of Biology, Faculty of Science, Chiang Mai University, Chiang Mai, Thailand; 2 Department of Biology, Faculty of Science, Chiang Mai University, Amphur Muang, Chiang Mai, Thailand; 3 Graduate School of Human Development and Environment, Kobe University, Kobe, Japan

**Keywords:** Clown featherback, Lake, Detection, eDNA based survey, Molecular tool

## Abstract

**Background:**

The importance of the inland fisheries sector in food security as a provider of much-needed protein and income supplier has been highlighted. This is especially the case in poor rural communities in developing countries. Inland capture fisheries in Thailand are in place nationwide in rivers, lakes, swamps and reservoirs. The clown featherback (*Chitala ornata*) is popularly consumed and is an economically important fish in Thailand which is often used in food products such as fish balls and fish cakes. Along with other fish species, the clown featherback is one of fish of inland fisheries at Phayao Lake. Recent fish surveys from 2016-2018 at Phayao Lake using netting and electrofishing found that the number of clown featherback have been reducing since 2016 and could not be detected at all by 2018. This is despite the fact that there are still reports of their presence in the lake from locals.

**Methods:**

We developed an eDNA-based method for detection of the clown featherback in Phayao Lake as an alternative tool. Water samples were collected in three different sampling months (February, June and September) at six sites located in the lake. Species-specific primers and the probe were designed to amplify a 183 bp fragment of the *cytB* region of the clown featherback.

**Results:**

eDNA of the clown featherback can be detected in all different sampling months and sites. Concentration of the clown featherback found in Prayao Lake showed no difference over sampling month but between collecting sites. This proves that eDNA based survey is a sensitive and useful tool for monitoring and surveying the clown featherback at any time of the year.

## Introduction

The clown featherback (*Chitala ornata*, Notopteridae) is a freshwater fish, native to tropical Asia, mainly found in the Mekong and Chao Phraya basins of Indochina and Thailand (Roberts, 1992) and Laos (Baird et al., 1999). Large individuals of this range of variety are often seen for sale for consumption at local markets. The clown featherback also features prominently in the aquarium trade. Fish is the primary source of animal protein for most people in Thailand. In 2016, fish consumption was 33.73 kg/capita/year, the highest consumed protein among all other animal protein sources such as pork, beef, and chicken. The clown featherback is popularly consumed and is one of the economically important fish in Thailand which, it is often used in food products such as fish balls and fish cakes (Vidthayanon, 2012; Uk-katawewat, 2004; Department of Fisheries, 2017).

Thai fisheries are characterized by their diversity of species and habitats, as well as their diverse inland fisheries. The importance of the inland fisheries sector in food security is that it provides much-needed protein and is an income supplier. This has been highlighted especially in poor rural communities of developing countries. (Oopatham, 2003). Inland capture fisheries in Thailand are carried out nationwide in rivers, lakes, swamps and reservoirs. These fisheries are an important sector of many local economies and are considered to be important in sustaining the livelihoods of many rural communities. Kwan Phayao or Phayao Lake is one of major lakes for inland fisheries in Thailand. In a total area of 19.8 km^2^, around 50 fish species (17 families) were reported in the Phayao Lake (Rattanadaeng, Panboon & Soe-been, 2015). Along with other fish species, the clown featherback is one of the main fish of inland fisheries at Phayao Lake. The Inland Aquaculture Research and Development Division (IARDD), Thailand routinely carries out surveys of fish species in the Phayao Lake. A combination of netting and electrofishing is the current method used to monitor fish species. The main limitations of this method are the varying efficiency of its tools (netting or electrofishing), incomplete samplings, not to mention the fact that it is time-consuming. Seasonal activity or behavior has had an influence on detection probabilities in traditional sampling approaches to measure the abundance or presence/absence of organisms (MacKenzie et al., 2002; Canessa et al., 2012). The survey team of IARDD reported that fish species are caught during different months of the year and their sampling sizes are different (Inland Fisheries Research and Development Division, 2019). Notable, none of them can be found in some years of the surveys based on traditional approaches despite the fact that locals reported their presence in the lake. Traditional samplings are labor intensive and limited in their reliability. The efficiency of each technique depends on the targeted species size and developmental stage (Jackson & Harvey, 1997; Lintermans, 2015) and different fishing tools may introduce different degrees of reliability in fish surveys (e.g., Porreca et al., 2013; Tate et al., 2003; Hanchin, Willis & St. Sauver, 2002). Thus, a more sensitive and reliable survey method is needed.

Advanced molecular techniques facilitate the estimating and monitoring of biodiversity, especially the increasing application of environmental DNA (eDNA). eDNA can be defined as short DNA fragments that organisms leave behind in environments (i.e., water, soil, or air). It holds great promise for monitoring both invasive and imperiled species and has proven to be a sensitive, effective and convenient method with increased speed. Recently the use of eDNA was proved to be a powerful tool in biodiversity science and conservation action, especially for fish species. For example, eDNA was used to evaluate the reintroduction program and to monitor the reintroduced fish species (e.g., Hempel et al., 2020; Riaz et al., 2020), to track distribution of endangered fish or those in difficult to access habitats (e.g., Laramie, Pilliod & Goldberg, 2015; Itakura et al., 2019), to gain more understanding about various ecological aspects such as breeding season, spawning activity, reproductive migration, and habitat (e.g., Takeuchi et al., 2019; Sakata et al., 2017; Antognazza et al., 2019; Thalinger et al., 2019), to detect invasive species (e.g., Nevers et al., 2018; Balasingham et al., 2018; Bellemain et al., 2016; Boothroyd et al., 2016) and design management strategies (e.g., Levi et al., 2019; Lacoursière-Roussel et al., 2016). Here, we developed an eDNA-based method for detecting the clown featherback in Phayao Lake as an alternative tool fore more effective, sensitive and less time-consuming survey which could be useful for fishery management.

## Materials & Methods

### Species-specific primers design

All the DNA tissue analyzed originated from the mucus of the individual clown featherback and tested species. The total DNA was extracted from the mucus sample using the Qiagen DNeasy kit (Qiagen, Valencia, CA). Extracted DNA was used as a template for qPCR assay together with synthetic fragments. DNA samples were quantified using a Qubit fluorometer (Life Technologies) calibrated with the Quant-iT dsDNA HS Assay following the manufacturer’s instructions. For each replicate, 3 µL volumes were measured.

Species-specific primers and a minor-groove binding (MGB) probe incorporating a 5′ FAM reporter dye and a 3′ non-fluorescent quencher were designed to amplify an 183 bp targeting within the *cytB* region for the clown featherback (*Chitala ornata*), using Primer Express (V3.0, Life Technologies; Table 1). Probe and primer sequences were matched against the National Centre for Biotechnology Information (NCBI, http://www.ncbi.nlm.nih.gov/) nucleotide database with BLASTn (Basic Local Alignment Search Tool) to confirm the species’ specificity for the clown featherback in silico assays.

**Table 1:** Details of species-specific primers and the probe designed to amplify a 183 bp fragment of the *cytB* region of *Chitala ornata*.

**Primer name**	**Type**	**Length (bp)**	**Primer sequence 5′-3 ′**
*cytB -F*	Forward primer	25	AATAAGCTAGGAGGTGTTCTAGCTC
*cytB -R*	Reverse primer	21	GCATGCCAGTAGAAGACCCGT
*cytB* -*PR*	Probe	18	TCGCAGATATAATTATCC

The qPCR assay was deployed using Environmental Master Mix (Applied Biosystems) on mucus samples from the clown featherback, related and commonly found species in Thai freshwater environments including Giant featherback (Chitala lopis), Grey Featherback (Notopterus notopterus), Great snakehead (Channa aurolineatus), Jullien’s golden carp (Probarbus jullieni), Red tailed tinfoil (Barbonymus altus), Smith’s barb (Puntioplites proctozysron) and Striped snakehead (Channa striata) to ensure the species specificity to the qPCR assay. Also, qPCR assay was carried out using water collected from the tank contained several fish species except the clown featherback. In addition, eDNA qPCR assay for the clown featherback, a water sample collected from tank at Phayao Freshwater Aquarium (Phayao Inland Fisheries Research and Development Center) was known to have only the clown featherback was included as a positive control for the presence of amplifiable eDNA in water samples. The tank contains around 4.5 m^3^ of water with one individual clown featherback residing in the tank (the fish is about 30–40 cm in length).

All eDNA qPCR amplifications were performed in three replicates in a final volume of 20 µL, using 10.0 µL of 2 × TaqMan Environmental Master Mix 2.0 (Thermo Fisher Scientific), 2.0 µL of DNA template, 900 nM each of the F/R primers, and 125 nM of the probe. Samples were run under the following conditions: an initial 10 min incubation at 95 °C followed by 50 cycles of denaturation at 95 °C for 15 s and annealing/extension at 60 °C for 1 min. Negative controls with all PCR reagents but no template (three replicates) were run in parallel to assess potential contamination. The quantification cycle (Cq) was converted to quantities per unit volume using the linear regression obtained from the synthesized target gene standard curve (Integrated DNA Technologies Pte. Ltd., Singapore). The clown featherback eDNA concentrations were then reported as copies/mL. The limit of detection (LOD) and the limit of quantification (LOQ) were also measured using standard dilution series of synthesized target gene fragment with known copy numbers. The concentration of the standards was adjusted to 15,000, 1,500, 150, 15, and 1.5 copies per reaction with 12 technical replicates used for each of the dilution steps. The calculation of LOD and LOQ was done using published R script by Merkes et al. (2019).

### Aquarium experiment

We used an aquarium experiment to test the extent to which qPCR of water samples can detect eDNA of clown featherback. The adult clown featherback was obtained from the fish store and transported to a laboratory at Chiang Mai University. The clown featherbacks were then held in separate 120 L plastic holding containers in which the water was continuously filtered. The fish were fed frozen shrimp /commercially available flake fish food three times a week, and were held at 23 ± 1 °C. All procedures were conducted in accordance with the current laws in Thailand on experimental animals and were approved by the safety management committee for experiments of the Laboratory Animal Center, Chiang Mai University (project number 2561/FA-0001).

We evaluated the sensitivity of eDNA detection in the aquaria by conducting three aquarium experiments using plastic tanks (30 × 45 × 25 cm) filled with 120 L of aged-tap water. The water in the tanks was continuously aerated through a filter. In each experiment, the clown featherback were randomly assigned to the tanks (2 individuals per container). The water in the tanks was maintained at 23 ± 1 °C. We collected a 300 mL water sample from each tank at each time point (0, 3, 6, 12, 24, 48, 72, 96, 120, 144, and 168 h after removal of the fishes from the tanks) in triplicate.Collected water was filtered on a GF/F filter (0.7 µm Whatman International Ltd., Maidstone, UK). The eDNA from each sample solution was extracted using a Qiagen DNeasy Blood & Tissue kit (Qiagen, Hilden, Germany) in a final volume of 50 µL. To confirm the absence of the clown featherback eDNA in the water prior to the experiments, three tanks without clown featherback were prepared and another water sample was collected and treated as described above.

Real-time PCR was performed with the species-specific primers and probe set using a Rotor-Gene Q system (Qiagen, Hilden, Germany). The reaction conditions were the same as described in qPCR assay section. Three replicates were conducted for each sample including the negative PCR control and positive control.

### eDNA field collection

**Figure 1:**
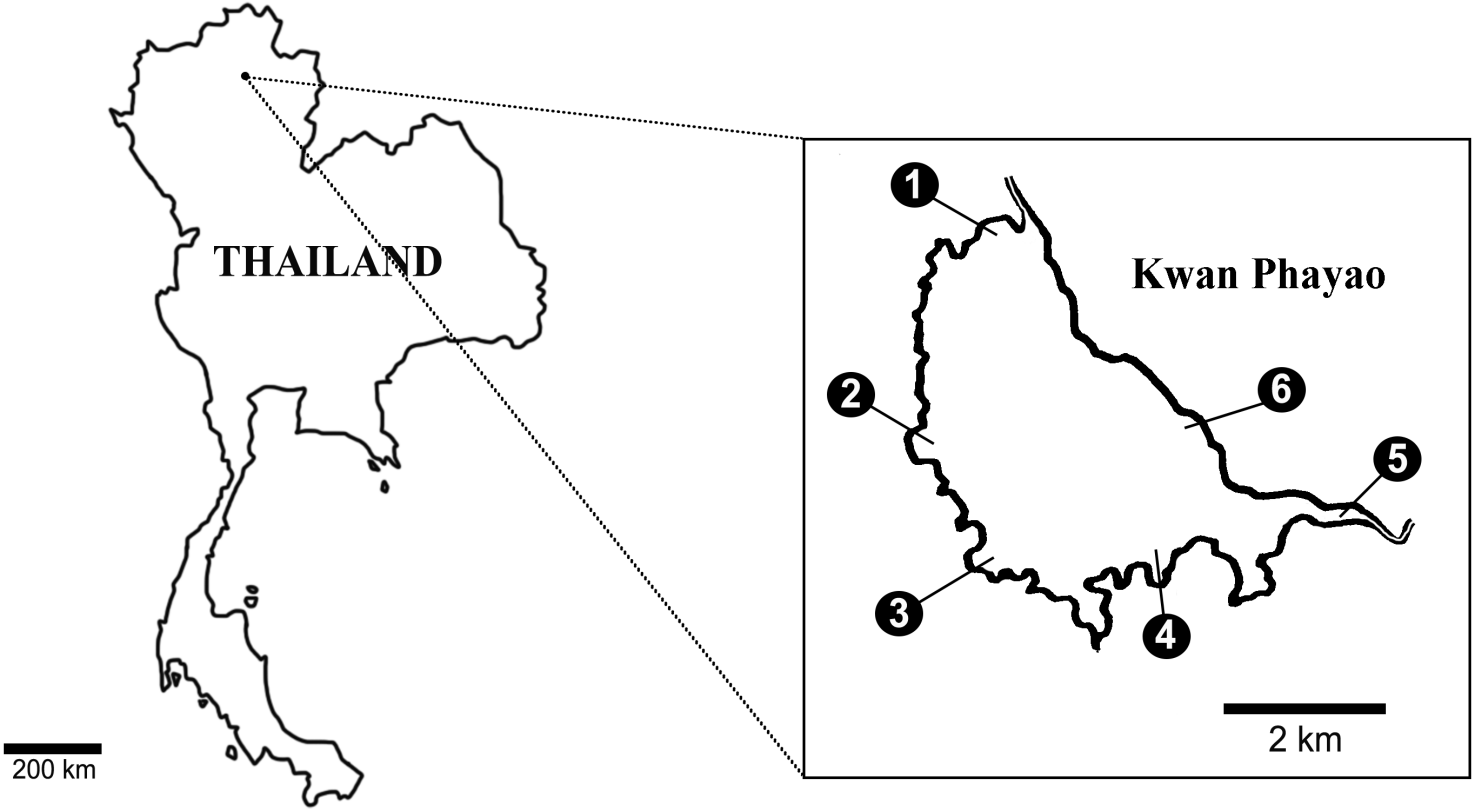
Six collecting sites in Payao Lake (Kwan1-Kwan6).

Water samples were collected at six points: within Payao Lake in September 2018; at the end of the rainy season; in February 2019; the middle of the dry season; and again at the beginning of rainy season in June 2019 (Fig. 1). Water samples were collected from the surface water of the lake. Each site was sampled in triplicate and 300 mL samples of water were collected and filtered on GF/F filter (0.7 µm Whatman International Ltd., Maidstone, UK). For every sampling day, deionized water (300 mL) was filtrated as a negative control. The water samples and real-time PCR were processed as described above. Statistical analyzes (Factorial ANOVA) were performed with R (R Core Team, 2017). Boxplots were produced to visualize the data using the ‘ggplot2’ package (Wickham, 2016).

### DNA extraction from the filters

DNA trapped on the filters obtained from the aquarium experiments and field collections were extracted using Qiagen DNeasy Blood and Tissue Kit (Qiagen, Hilden, Germany) using a protocol modified from the manufacturer’s protocol with the following changes: the DNA from all samples were eluted twice with 25 µL AE buffer, in a total volume of 50 µL to obtain a more concentrated eDNA solution. The volume of ATL buffer (360 µL), Proteinase K (40 µL), AL buffer (400 µL) and Ethanol (400 µL) were doubled.

## Results

Here, we designed primers that amplify short target regions which is 183 bp specific the clown featherback (*C. ornata*). To determine specificity, the designed primers were tested in silico. The specificity of the generated primers was checked against the NCBI database by comparing of similarity of primers to *Chitala* and related species. The homology of *Chitala* sequences to the specific forward and reverse primers of *C. ornata* is 82.6%–87.0% with at least five mismatches on the forward and four mismatches on the reverse primers (Table 2). When compared with other fish species commonly found in Phayao Lake, the homology was found to be lower (69.6%–78.3%). In addition, all the qPCR tests for the primers’ specificity of DNA extracted from other fish and water collected from the tank with mix fish species (without the clown featherback) were negative.

**Table 2:** Homology of the query to the forward and reverse primers, percentage identity as a function of the number of matching base sites divided by 45 (total number of base sites across the primer pair) Base site homology between the query and the primer is shown as a dot.

**Species**	**Forward**	**Reverse**	**Identity, %**	**GenBank**
*Chitala ornata*	•••••••••••••••••••••••••	••••••••••••••••••••	100.0	AB035243
*Chitala blanci*	••C••A••••••••C••••••••C•	••••A•••••••••••••A•	87.0	AP008921
*Chitala lopis*	••C•••••••••••C••C•••••C•	•T••A••••••••A••••A•	82.6	AP008922
*Notopterus notopterus*	••C••A••T•••••C••C•••••C•	••••A•••••••••••••A•	82.6	AP008925
*Cyprinus carpio*	••C••A••T•••••••••C••T••A•	••••A••••••••C•T••C•	78.3	DQ868875
*Hampala macrolepidota*	••C••A••C•••••A••C••T••C•	••••A••••••••C••••A•	78.3	KC696545
*Helostoma temminckii*	••C••A••C••C••A••C••G••C•	•A••A••T••C•••A•••C•	69.6	AY763742
*Monopterus albus*	•GC••A•••••C••A••AA•T••C•	••••A••T•••••T••••A•	71.7	KX155542
*Pristolepis fasciata*	••C••A••T••G••C••C•••••C•	•A••••C••T•••C••••C•	73.9	KR131450
*Puntius brevis*	•••••AT••••G••A••T••T••A•	•T••A••••••••C••••A•	76.1	HM536815
*Rasbora argyrotaenia*	••C••A••••••••A••T••T••A•	•A••A•••••••GC••••A•	76.1	HM224335
*Trichopsis vittata*	••C••A••••••••A••T••C••C•	•T••A••C•••••C••••A•	73.9	AF519697
*Trichogaster pectoralis*	••C••A••T•••••A••G•••••C•	•A•••••CC•T•••••••T•	76.1	AY763758

Sensitivity of the qPCR assay is expressed as the limit of detection (LOD) with 95% of confidence and the limit of quantification (LOQ) with a threshold of ≤ 35%. The qPCR assay of the clown featherback had a LOD = 9.8 copies per µL and LOQ = 9.8 copies per µL. We also used an aquarium experiment to test the extent to which the qPCR of water samples can detect the clown featherback. We were able to detect the eDNA of the clown featherback from water samples collected at time 0, 3, 6, 12, 24, 48, 72, 96, 120, 144, and 168 h, after removing the fishes. Thus, the eDNA qPCR assay resulted in positive DNA signals in 100% of the aquarium experiment even 7 days after the removal of the fishes from the tanks.

**Figure 2:**
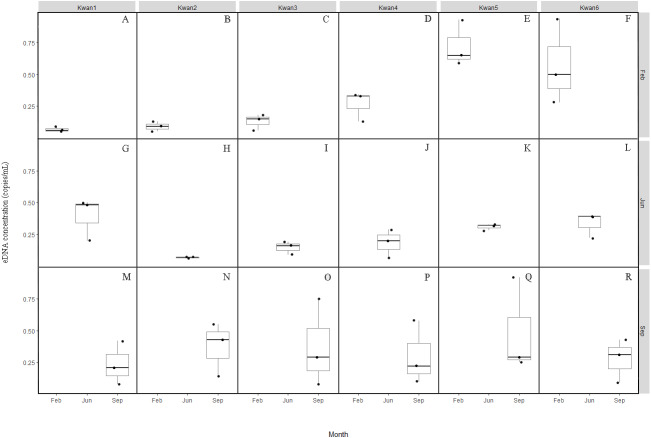
Box plots of the clown featherback eDNA concentration (copies/mL) across the three sampling month February (A–F), June (G–L) and September (M–R).

Previous traditional surveys of fish in Phayao Lake failed to recognise the presence of the clown featherback in some seasons and years even though reported by locals who had found and caught them from the lake.. We collected water samples from six sites at the same locations where the teams from the Inland Fisheries Research and Development Division, Department of Fisheries conducted the traditional surveys (Fig. 1). Three different sampling months (February, June and September) were compared. The eDNA of the clown featherback were detected in water samples from all collecting sites in Phayao Lake of all three sampling months (Table 3 and Fig. 2). The efficiency for the qPCR assay was 91% (*y* =  −3.549x + 40.397; *R*^2^ = 0.98). These regression equations were used to convert the quantification cycle (Cq) data from the qPCR product (i.e., the PCR cycle at which the target is considered positively amplified in a given sample) to the concentration of DNA in a given sample (copies of DNA per unit volume).

**Table 3:** eDNA concentration (copies/mL) of the clown featherback found in Prayao Lake.

Site	Replication	Month		
		September	February	June
Kwan1	1	0.42	0.05	0.50
Kwan1	2	0.00	0.09	0.48
Kwan1	3	0.08	0.06	0.39
Kwan2	1	0.43	0.05	0.07
Kwan2	2	0.14	0.09	0.07
Kwan2	3	0.55	0.13	0.06
Kwan3	1	0.08	0.06	0.19
Kwan3	2	0.29	0.15	0.09
Kwan3	3	0.75	0.18	0.16
Kwan4	1	0.22	0.13	0.06
Kwan4	2	0.10	0.33	0.29
Kwan4	3	0.58	0.34	0.20
Kwan5	1	0.29	0.59	0.28
Kwan5	2	0.25	0.65	0.32
Kwan5	3	0.92	0.93	0.33
Kwan6	1	0.31	0.28	0.22
Kwan6	2	0.09	0.50	0.39
Kwan6	3	0.43	0.94	0.39

Concentration of the clown featherback found in Prayao Lake ranges from around 0.05 –0.94 copies/mL in February, 0.06–0.50 copies/mL in June, and 0.08–0.92 copies/mL in September with no difference over sampling month (*P* = 0.42). In contrast, we found a significant difference (*p* = 0.005) in measured eDNA concentrations between the collecting sites, an average eDNA concentration of 0.23 copies/mL at Kwan1 (range: 0.05–0.50), 0.18 copies/mL at Kwan2 (range: 0.05–0.55), 0.22 copies/mL at Kwan3 (range: 0.06–0.75), 0.25 copies/mL at Kwan4 (range: 0.06–0.58), 0.51 copies/mL at Kwan5 (range: 0.25–0.93), and 0.40 copies/mL at Kwan6 (range: 0.09–0.94). The findings show that eDNA based survey is one of a few powerful tools for monitoring and surveying the clown featherback at any time of the year.

## Discussion

Overexploitation of a fishery may not simply be marked by species being undetected during sampling in which may resulting from insensitivity and unreliable of traditional survey methods. Recently, several studies have already successfully shown eDNA sampling can be more sensitive and required lower sampling effort than traditional approaches using physical capture in several instances (e.g., Jerde et al., 2016; Takahara, Minamoto & Doi, 2013; Laramie, Pilliod & Goldberg, 2015; Smart et al., 2015; Bista et al., 2017; Hinlo et al., 2017; Evans et al., 2017). eDNA method holds great potential of overcoming many limitations of traditional sampling methods, although there are some challenges. Breeding and migratory seasons are factors that influence eDNA detectability and should be considered in eDNA surveys (Spear et al., 2015; Barnes & Turner, 2016; Buxton et al., 2017). However, we did not find seasonal dynamics in eDNA concentration of the clown featherback in this study. The average eDNA copies detected from all collecting sites did not significantly different over the sampling months. On the other hand, eDNA signals of each sampling site (Kwan1-Kwan6) were found to be different. The highest average eDNA copies of the clown featherback were found at Kwan5. Similarly, Kwan5 is the sampling station where fish abundance is the highest in Phayao Lake (Rattanadaeng, Panboon & Soe-been, 2015). The variation of eDNA detection between sampling sites within the lake could be due to an uneven (patchy) distribution of organisms which is commonly found in the lentic system, thus leading to higher concentrations of eDNA in areas where organisms were present (Takahara et al., 2012; Eichmiller, Bajer & Sorensen, 2014).

In this study, although, experimentation on the relationship between organismal density and eDNA concentration was not carried out, there are several previous studies which indicated positive correlations between biomass or abundance and eDNA quantities (e.g., Takahara et al., 2012; Maruyama et al., 2015; Lacoursière-Roussel, Rosabal & Bernatchez, 2019; Itakura et al., 2019). Also, eDNA detection probability was observed to increase with specimen density (Stoeckle et al., 2017).

In addition, we cannot rule out that environmental conditions such as temperature, light and pH impact eDNA concentration of target species (Review in Stewart, 2019; Harrison, Sunday & Rogers, 2019).

## Conclusions

Recently, the use of environmental DNA (eDNA) has proved to be a powerful tool for fish survey or sampling. eDNA surveying is likely to have a major impact on the ability of species detection. The ability to rapidly and sensitively detect the presence of a target species through eDNA analysis has enabled a wide range of scientific discoveries and technological advancements. To the best of our knowledge, this is the first report of eDNA detection of the clown featherback in Thailand. Taken together, these findings suggest the eDNA method is an effective tool for targeted species detection to complement traditional sampling/survey approaches and is one of molecular methods which useful for assessment the presence of a fish species in a confined environment such as a lake.

##  Supplemental Information

10.7717/peerj.10338/supp-1 Supplemental Information 1 qPCR data generated from water samples collecting in September, February and June There are three replications (A, B, C) from six sampling sites (Kwan1-Kwan6).

## Additional Information and Declarations

### Data Availability

The following information was supplied regarding data availability: qPCR data generated from water samples collecting in September, February and June are available in the Supplemental Files. R code from Merkes et al. (2019) was used to calculate LOD and LOQ. Merkes CM, Klymus KE, Allison MJ, Goldberg C, Helbing CC, Hunter ME, Jackson CA, Lance RF, Mangan AM, Monroe EM, Piaggio AJ, Stokdyk JP, Wilson CC, Richter C. 2019 Generic qPCR Limit of Detection (LOD) / Limit of Quantification (LOQ) calculator, R Script, U.S. Geological Survey code release, https://doi.org/10.5066/P9GT00GB.
